# Alterations in the sense of time, space, and body in the mindfulness-trained brain: a neurophenomenologically-guided MEG study

**DOI:** 10.3389/fpsyg.2013.00912

**Published:** 2013-12-03

**Authors:** Aviva Berkovich-Ohana, Yair Dor-Ziderman, Joseph Glicksohn, Abraham Goldstein

**Affiliations:** ^1^Department of Neurobiology, Weizmann Institute of ScienceRehovot, Israel; ^2^The Leslie and Susan Gonda (Goldschmied) Multidisciplinary Brain Research Center, Bar-Ilan UniversityRamat Gan, Israel; ^3^Department of Criminology, Bar-Ilan UniversityRamat Gan, Israel; ^4^Department of Psychology, Bar-Ilan UniversityRamat Gan, Israel

**Keywords:** space perception, time perception, body perception, magnetoencephalography (MEG), theta rhythm, neurophenomenolgy, mindfulness meditation

## Abstract

Meditation practice can lead to what have been referred to as “altered states of consciousness.”One of the phenomenological characteristics of these states is a joint alteration in the sense of time, space, and body. Here, we set out to study the unique experiences of alteration in the sense of time and space by collaborating with a select group of 12 long-term mindfulness meditation (MM) practitioners in a neurophenomenological setup, utilizing first-person data to guide the neural analyses. We hypothesized that the underlying neural activity accompanying alterations in the sense of time and space would be related to alterations in bodily processing. The participants were asked to volitionally bring about distinct states of “Timelessness” (outside time) and “Spacelessness” (outside space) while their brain activity was recorded by MEG. In order to rule out the involvement of attention, memory, or imagination, we used control states of “Then” (past) and “There” (another place). MEG sensors evidencing alterations in power values were identified, and the brain regions underlying these changes were estimated via spatial filtering (beamforming). Particularly, we searched for similar neural activity hypothesized to underlie both the state of “Timelessness” and “Spacelessness.” The results were mostly confined to the theta band, and showed that: (1) the “Then”/“There” overlap yielded activity in regions related to autobiographic memory and imagery (right posterior parietal lobule (PPL), right precentral/middle frontal gyrus (MFG), bilateral precuneus); (2) “Timelessness”/“Spacelessness” conditions overlapped in a different network, related to alterations in the sense of the body (posterior cingulate, right temporoparietal junction (TPJ), cerebellum); and (3) phenomenologically-guided neural analyses enabled us to dissociate different levels of alterations in the sense of the body. This study illustrates the utility of employing experienced contemplative practitioners within a neurophenomenological setup for scientifically characterizing a self-induced altered sense of time, space and body, as well as the importance of theta activity in relation with these altered states.

## Introduction

Long-term contemplative practitioners offer an exclusive opportunity to study unique mental states, due both to their heightened introspective abilities, as well as their ability to intentionally alter subtle aspects of consciousness (Lutz et al., 2007). Here, we employ long-term Mindfulness meditators to study unique states of alteration in the sense of time and space, which have not yet been neuroscientifically investigated.

One of the characteristics of altered states of consciousness is a *joint* alteration in the experience of time and space (Tart, 1972; Glicksohn, 1993; Baruss, 2003; Glicksohn and Berkovich-Ohana, 2011), which has been called by Fingelkurts and Fingelkurts (2006) “a sense of timelessness, spacelessness.” The incidence of mutual alteration in the experience of time and space is so common that it led Walter Stace, the well-known scholar of mysticism, to include one characteristic named “non-spatial and non-temporal” (1960, p. 110) in his definition of the universal core of mystical experience. According to Suzuki, *sunyata*, the Buddhist concept of emptiness, means: “absolute emptiness transcending all forms of mutual relationship… There is no time, no space, no becoming, nothingness… when the mind is devoid of all its possible content” (in Stace, 1960, p. 109). Similarly, in Vedic psychology, transcendental consciousness, which is a state achieved through the practice of Transcendental Meditation in which the individual's mind transcends all mental activity to experience the simplest form of awareness, is characterized by being unbounded in space and time (Alexander et al., 1987).

Cognitively, an altered sense of time can be viewed as the limit for the functioning of the cognitive timer, the breakdown of apparent duration (Glicksohn, 2001). Apparent duration, in turn, is closely related to spatial perception (Boroditsky and Ramscar, 2002; Glicksohn and Myslobodsky, 2006; Srinivasan and Carey, 2010; reviewed by Walsh, 2003). Neuroscientific evidence suggests common underlying mechanisms for spatio-temporal processing (Basso et al., 1996; Walsh, 2003; Danckert et al., 2007; Oliveri et al., 2009a), as do linguistic (Boroditsky and Ramscar, 2002; Núñez and Sweetser, 2006; Casasanto, 2008, 2010) and psychophysical studies (e.g., Sarrazin et al., 2004; Oliveri et al., 2009a,b; Srinivasan and Carey, 2010). An altered experience of space has been called by Stanley (1898) “space annihilation,” again in relation to the time dimension. An altered sense of time and space has hardly been studied scientifically, the major obstacle being the production of these experiences in the lab (but see the hypnosis experiment of Aaronson, 1970).

Phenomenologically, altered states of consciousness are frequently accompanied by a joint alteration in both the experience of time and space, as noted above, but also in bodily perception (Tart, 1972; Travis and Pearson, 2000; Shanon, 2003; Vaitl et al., 2005; Hunt, 2007; Ataria and Neria, 2013). Hence, we hypothesized that an altered sense of time and space would be related to an altered sense of body. An altered sense of body is conceptualized as a disrupted sense of spatial unity between self and body, where the self is not experienced as being confined within the boundaries of the body. A possible candidate mediating this connection is the insula, related to both proprioception and the sense of time (Craig, 2002, 2009a,b).

Mindfulness meditation (MM) practice focuses on cultivating a non-judgmental awareness of momentary experience (Kabat-Zinn, 2003) with the aim of liberation from human suffering (Olendzki, 2003; Dreyfus and Thompson, 2007). While not being the goal of training, long-term practice is often accompanied by altered states of consciousness (Stace, 1960; Goleman, 1988; Shapiro, 2008). This makes MM practitioners potentially familiar with alteration in the experience of both time and space. In addition, advanced practitioners have been documented as not only being able to produce voluntary alterations of subtle consciousness-related experiences within laboratory settings, but also to provide refined first-person descriptions of these experiences (Lutz et al., 2007). This renders MM practitioners ideal candidates for the study of such unique states, which have hitherto been quite unexplored.

Here, we study the experience of alteration in the sense of time and space by collaborating with a select group of 12 long-term MM practitioners in a neurophenomenological setup. All of the participants have experienced these states in the past (see Methods), and thus possess a frame of experiential reference. The participants were asked to volitionally produce two distinct states, which we *a priori* named “Timelessness” and “Spacelessness” (outside time and space, respectively). In order to rule out the involvement of attention, autobiographic memory or imaginative processes (Szpunar et al., 2007), participants were also asked to produce control states of “Then” and “There” (be in the past and in another place, respectively). The data were analyzed in terms of “Timelessness” vs. “Now” and “Spacelessness” vs. “Here,” and contrasted with “Then” vs. “Now” and “There” vs. “Here” (two target and two control contrasts, respectively). In particular, we investigated whether there would be similar neural activity underlying both these states of “Timelessness” and “Spacelessness.” Specifically, we hypothesized that: (1) the control conditions, “Then” and “There,” would yield overlapping activity in an autobiographic memory network; (2) “Timelessness” and “Spacelessness” conditions would overlap in a different network, related to alterations in the sense of body, including the insular cortex; and (3) phenomenologically-guided neural analyses would yield further insight into the underlying physiology of the alteration in the sense of time and space.

## Methods

### Participants

Sixteen practitioners participated in this study, of whom two were excluded due to self-reported severe tiredness and back pain, respectively, during the data recordings. Two others were excluded as they practiced different forms of meditation (not MM), in an attempt to homogenize the group. The remaining participants practice within the Theravada tradition. Participants were right-handed (3 females, age 44.9 ± 10.9 years, range: 31–64) and healthy, with no history of mental or neurological diseases. All participants were long-term practitioners with an average of 16.5 (*SD* = 7.9, range: 9–34) years, and 11,225 (*SD* = 9909, range: 1290–29,290) total hours, of meditation practice. The study was approved by the Research Ethics Board of Bar-Ilan University. The participants gave their written consent and were financially compensated.

### Pre-recording procedures

The participants were introduced to the lab, then filled out forms (research consent, personal details, formal practice estimate) and Hood's (1975) Mysticism scale, to test for previous experiences of alteration in the sense of time and space. The experimenters explained to the participants each part of the experiment, and it was made certain that the participants understood the tasks. Altogether, this part took 45–60 min.

### Hood's (1975) mysticism scale (Mscale)

Hood's (1975) Mscale is a general measure of self-transcendent experience, based on Stace's (1960) conceptualization of eight dimensions of transcendence. This is a 32-item scale, where the items are grouped into eight components of experience: *Positive affect, Religious quality, Noetic quality, Ineffability, Unifying quality, Inner subjective quality, Ego quality*, and *Temporal and spatial quality*, the last being an experience of “timelessness” and “spacelessness.” The results of the last item were used to assess whether participants had previous experiences of altered sense of time and space, of interest for this report. The four statements pertaining to this item were: (1) I have had an experience which was both timelessness and spacelessness; (2) I have had an experience in which I had no sense of time or space; (3) I have never had an experience in which time, place, and distance were meaningless; and (4) I have never had an experience in which time and space were non-existent. Participants were requested to indicate on a five-point scale from −2 (*definitely not true*) to +2 (*definitely tru*e), the extent to which each of 32 statements is true of their own experiences. After reversing appropriate items, these responses are converted to a five-point Likert scale, from 1 (*low*) to 5 (*high*), where indecision is scored as 3.

The mean score for the Mscale was 4.30 ± 0.40, indicating that participants experienced mystical states high above the indecision point. Importantly, mean score for the sub-scale of *Temporal and spatial quality* was high, 4.47 ± 0.67. Thus, participants had previous acquaintance with the concurrent experience of alteration in the sense of time and space. For comparison, the mean Mscale score in a population of 191 religious participants was 3.58, and the *Temporal and spatial quality* was 3.42 (Lazar and Kravetz, 2005). This indicates that meditation practice increases the occurrence of alteration in the sense of time and space experiences high above mere religious tendency.

### Experimental tasks

The experiment comprised seven MEG recording sessions. Each session was followed by an interview conducted via the intercom system, during which brain activity was not recorded. The participants were encouraged to stretch their limbs and relax during the interviews, but were requested not to move and to keep their eyes closed while performing the tasks. To correct for head and body movements during the interviews, head-shapes were re-registered at the beginning of each session. A 20-min break was suggested to the participants after completing the 5th session of the experiment, during which refreshments were offered. Total time in the MEG was around 2 h.

**Figure 1:**
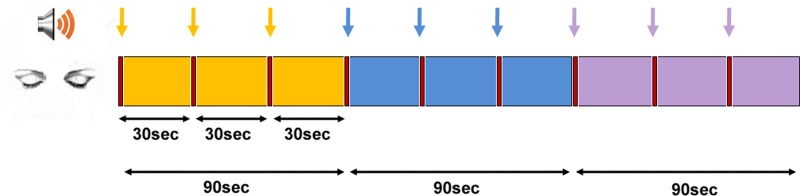
**Experimental protocol**. The time and sequence of conditions in the two MEG sessions of “time” and “space.” Yellow—“There”/“Then,” blue—“Here”/“Now,” and purple—“Timelessness”/“Spacelessness” conditions. All epochs were initiated by an auditory cue (marked by arrows).

The two sessions reported here are the “Time” and “Space” sessions (3rd and 4th, respectively), which were preceded by a resting state and a time production session, and followed by a “Self” session, reported elsewhere (Dor-Ziderman et al., 2013). The participants were asked to volitionally bring about alterations in their experience of time and space, in a manner which had been previously explained. Each of the two sessions comprised 3 conditions, each repeated 3 times in succession for 30 s (Figure 1). A recording with instructions for each condition was sounded (<2 s), after which the participant performed the requested task for 30 s. At the end of the 30 s, a short sound was heard indicating the participant to stop, and then the next instruction was delivered. The session was followed by a structured interview conducted via the intercom system.

The specific instructions for the three conditions in the “Time” session were:

“Now”—“Try to be in the present moment”
“Then”—“Try to be in the near past (in the same place—the lab)”
“Timelessness”—“Try to be outside time”

The specific instructions for the three conditions in the “Space” session were:

“Here”—“Try to be here”
“There”—“Try to be elsewhere (at the moment, with the experimenters outside the shielded MEG room)”
“Spacelessness”—“Try not to be in the center of space”

### Subjective reports

#### Phenomenological analyses

Participants were asked, immediately after each session, to describe their experiences during the session, following a semi-structured interview. The reports of the “Timelessness” and “Spacelessness” conditions were carefully analyzed by the first author. Two broad themes emerged: “sense of time and space,” and “bodily boundaries,” each including several categories of experience. Subsequently, participants were allocated into one of three categories for the “sense of time and space” theme, and into one of four categories for the “bodily boundaries” theme. The reports were then given to four other judges for anonymous rating. A classification of any report to one of the categories was accepted based on the majority (minimum three out of five) (Table 1).

**Table 1:**
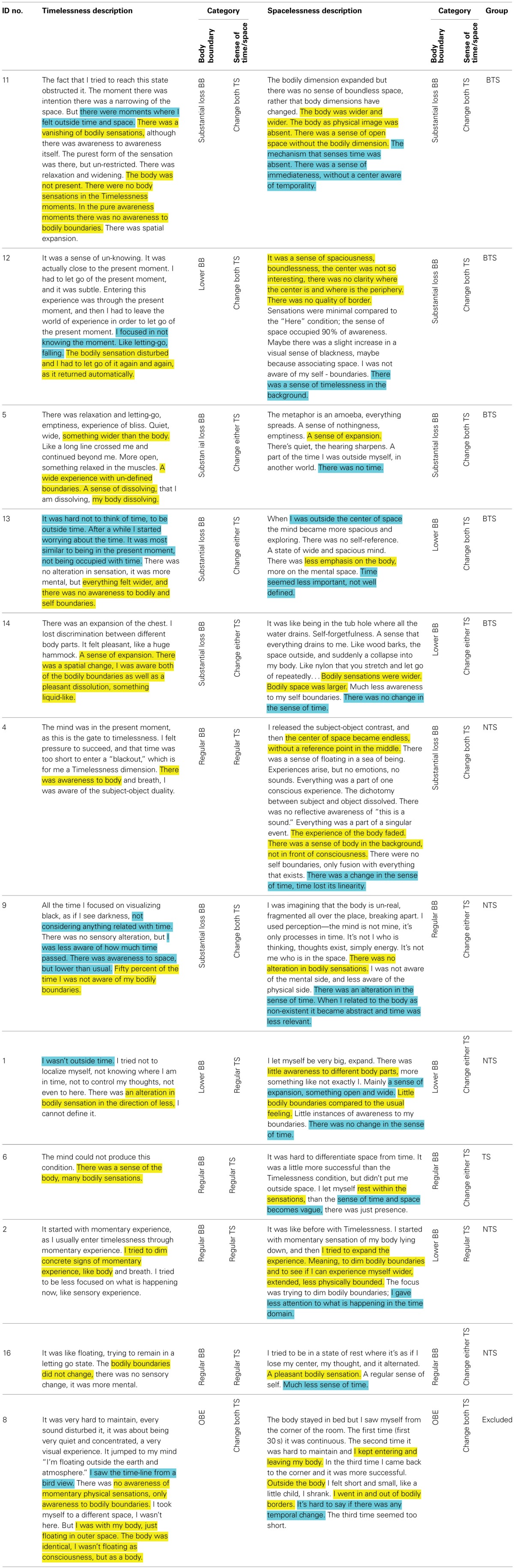
**Classification of participants to phenomenological categories and groups**.

Yellow and cyan, sentences pertaining to body or to time/space, respectively. BB, bodily boundaries; OBE, Out of body experience; TS, time and space; BTS, Both time and space; NTS, Not both Time and Space.

For the “sense of time and space” theme, three categories emerged, all along a continuum of increasing alteration in the sense of time and space:

- Regular sense of time and space (regular TS)—regular experience of time and space.
- Change in the sense of either time or space (Change either TS)—an alteration in the usual sense of time or of space.
- Change in the sense of both time and space (Change both TS)—an alteration in the usual sense of both time and space.

For the “bodily boundaries” theme, four categories emerged, the first three along a continuum of alteration in bodily boundaries and egocentric frame of reference:

- Regular bodily boundaries (regular BB)—regular experience of bodily boundaries, fully egocentric experience.
- Lower bodily boundaries (lower BB)—weaker than usual experience of bodily boundaries, bodily expansion, a reduction in the egocentric experience.
- Substantial loss of bodily boundaries (Substantial loss BB)—very weak, and occasionally a total loss of experience of bodily boundaries, strong bodily expansiveness, a strong reduction in the egocentric frame of reference.
- Out-of-body experience (OBE)—an extracorporeal egocentric perspective (floating outside one's body and perceiving one's physical body from a place outside one's body), with normal or distorted bodily boundaries.

In line with this analysis, 11 participants could be placed along a continuum of alteration in bodily boundaries and egocentric frame of reference. As the OBE could not be placed along the continuum of reduction in egocentric frame of reference, the sole participant having this experience was excluded from all subsequent analyses (see Table 1). The remaining 11 participants were included in the subsequent analyses, and were regarded as being placed along one continuum: at one end were those who experienced a weak and gentle shift, while at the other end were those who experienced a profound shift. This is in line with Shapiro (2008) who states that unique meditative states “need to be seen along a continuum. On one hand of the continuum are “full blown” mystical experiences, at the other, more common alterations of perception” (p. 25). Next, we set out to differentiate between participants along the higher and lower ends of the above continuum, by creating two groups (Table 1):

1. Both Time and Space (BTS) group—during the two conditions of “Timelessness” and “Spacelessness,” participants experienced a change in both themes of phenomenal experience (i.e., do not belong to “Regular BB” or “Regular TS”).
2. Not both Time and Space (NTS) group—during the two conditions of “Timelessness” and “Spacelessness,” participants did not experience a change in both themes of phenomenal experience (i.e., belong to “Regular BB” or “Regular TS”).

Testing for differences between the BTS and NTS groups, there were no significant differences in age or practice experience, as well as mean score for the Mscale or the sub-scale of *Temporal and spatial quality*.

#### Success and stability ratings

**Figure 2:**
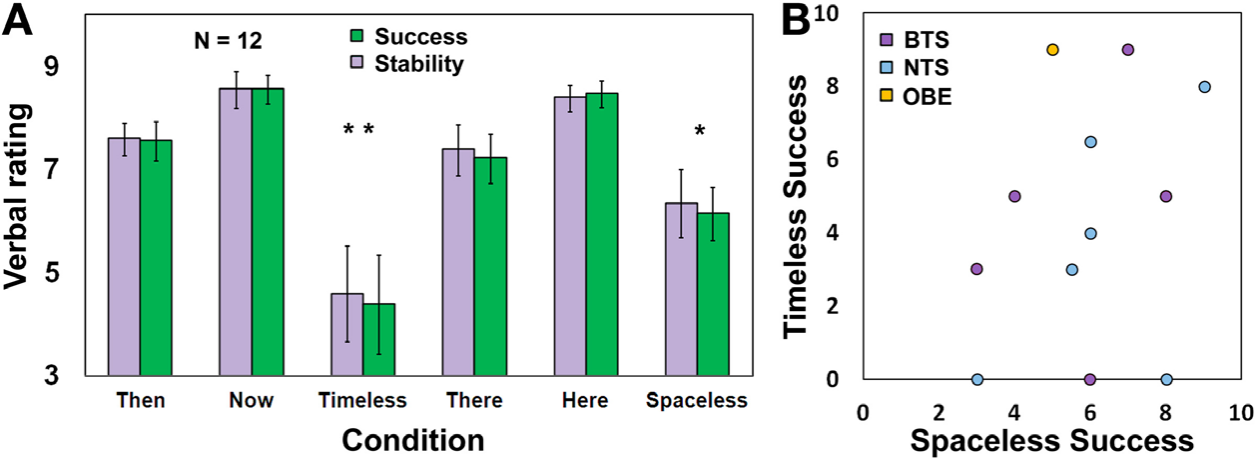
**Rating of task success and stability. (A)** Participants' rating (Mean ± s.e.m., *n* = 12) for success and stability during the different conditions. ^*^p < 0.05, ^**^p < 0.005, Bonferonni-corrected; **(B)** A scatterplot for the Timelessness vs. Spacelessness rated success. BTS, Both time and space; NTS, Not both Time and Space. Yellow dot refers to one OBE participant.

Participants were asked to verbally rate, on a 1–10 scale (1—“very low,” 10—“very high”), their success (defined here as: how strong was the experience, on average, across the 90 s allocated for each condition) and stability (defined here as: how stable was the experience during the 90 s allocated for each condition) in performing each of the tasks. Subsequently, a Three-Way ANOVA was conducted, with Session (Time/Space) × Condition (Then/There, Here/Now, Timelessness/Spacelessness) × Measure (success, stability) on these ratings. There was no difference between success and stability (Figure 2A), hence we will focus subsequently on success. We found only a main effect for Condition [*F*_(2, 20)_ = 18.5, *MSE* = 5.07, *p* <0.0001]. The “Timelessness” and “Spacelessness” conditions were rated as significantly less successful compared to “Here” and “Now” [*t* = 3.80, *p* = 0.011 and *t* = 3.99, *p* = 0.0045, respectively, Bonferroni-corrected *post-hoc* paired *t*-tests], but showed no significant difference between them. When comparing scores for the “Timelessness” and “Spacelessness” conditions between the BTS and NTS groups, no significant difference was found. Figure 2B depicts the rating for the “Timelessness” vs. “Spacelessness” conditions, showing the BTS and NTS groups to be similarly distributed. This is in contrast to the phenomenological difference found between the groups. We suggest that the discrepancy stems from different levels of self-criticism, as well as different personal expectations. In fact, some of the most-experienced practitioners scored their success as being lower compared to the less-experienced practitioners. Altogether, our results emphasize the need to collect verbal reports in addition to self ratings when conducting neurophenomenological analyses.

An additional observation is that the “Timelessness” scores were more variable compared to the “Spacelessness” scores. The reason for this is that two of the three participants reporting zero success in the “Timelessness” condition belong to the Burmese Mahasi school, which adheres strictly to the “Progress of insight” which is an inner “map” of how insights unfold through 16 developmental stages of insight knowledge. This tradition encourages an experience of “cuts” in consciousness, which are considered the culmination of these stages, and are called fruition (*phala*). Nanarama (1983) describes this state: “consciousness… transcends the continuous occurrence of formations and aligns upon non-occurrence” (p. 117). The fruition is mentioned here in some length, as it turned out that these two participants attempted to reach this acquainted state unsuccessfully during the short time allocated to the “Timelessness” condition, which affected their rating of success. However, after the Time session, it was emphasized that the experimenters did not expect these unique fruition states during the short lab procedure, thus their scores for the “Spacelessness” condition increased markedly, to 6 and 8.

### Magnetoencephalography (MEG)

#### MEG data acquisition

MEG recordings were conducted with a whole-head, 248-channel magnetometer array (4-D Neuroimaging, Magnes 3600 WH) in a magnetically-shielded room. Reference coils located ~30 cm above the head oriented by the *x*, *y*, and *z* axes were used to remove environmental noise. Head position was indicated by attaching 5 coils to the scalp and determining, to a 1 mm resolution, their position relative to the sensor array before and after measurement. Head localization was performed before and after each set of tasks to determine degree of head movement. Head shape and coil position were digitized using a Pollhemus FASTTRAK digitizer. Brain signals were recorded with a sampling rate of 1017.25 Hz and an analog online 0.1–400 Hz band-pass filter. The instructions for each condition were presented using E-prime 1.0 and delivered via a STAX SRS-005 amplifier and SR-003 push-pull electrostatic ear speakers coupled by a vinyl tube to silicon earpieces to prevent magnetic noise within the shielded room. Task performance ratings were collected using a LUMItouch photon control response box.

#### MEG data cleaning and preprocessing

Data processing and analysis was performed using Matlab® R2009b and FieldTrip toolbox for MEG analysis (Open source software for advanced analysis of MEG) (Oostenveld et al., 2011). Data were cleaned for line frequency (by recording on an additional channel the 50 Hz from the power outlet, and subtracting the average power-line response from every MEG sensor), and 24 Hz building vibration (measured in *x*, *y*, and *z* directions using 3 Bruel and Kjaer accelerometers) artifacts (Tal and Abeles, 2013). The data from the 3 “Time” and 3 “Space” conditions were then segmented into non-overlapping 2-s epochs. Each epoch was visually examined for muscle and jump artifacts (in the MEG sensors). Contaminated epochs were discarded. No malfunctioning MEG sensors were identified. To ensure the removal of all heartbeat, eye and muscle artifact, an independent component analysis (ICA) was performed on the data (Jung et al., 2000). Segmented data were down-sampled to 339 (1017/3) Hz to speed up data decomposition. The data were then decomposed into a set of independent components (248, equal to the number of sensors) ordered by degree of their explained variance. Components indicating heartbeats or eye movements were determined from a visual inspection of the 2D scalp maps and time course of each component. The remaining components were then used to reconstruct the pre down-sampled data.

#### Sensor-level analyses

The first 4 s of each task (3 tasks of 30-s in each condition) were omitted so as to allow the participants sufficient time to enter the states. This decision was made after consulting an expert meditator as to the study design, and following two self-pilot runs (with Aviva Berkovich-Ohana and Yair Dor-Ziderman, also long-term practitioners). From the remaining data in each condition, the first 32 epochs were used for further sensor-level analyses. These 2-s epochs were multiplied by a Hanning taper, and subjected to a Fast Fourier Transformation (FFT) for the frequencies ranging from 4 to 45 Hz. This resulted in a power spectrum with a frequency resolution of 0.5 Hz for each epoch. The power spectra were then averaged across the epochs of each condition and across the theta (4–8 Hz), alpha (8–13 Hz), beta (13–25 Hz), and gamma (25–45 Hz) frequency bands, thus obtaining mean power for each condition, participant and frequency band.

Sensor-level cluster-based statistics were assessed, and corrected for multiple comparisons, using a Monte-Carlo non-parametric permutations approach (Maris and Oostenveld, 2007). This approach was chosen as it does not make any assumptions on the underlying distribution. Finally, 2D *t*-value scalp topographies marking the significant clusters were created.

#### Source-space projection

Localization was performed for all frequency bands. Sources were estimated using Synthetic Aperture Magnetometry (SAM, Robinson and Vrba, 1999). SAM is an adaptive non-linear minimum-variance beamformer algorithm. It calculates the signal covariance from the MEG sensor data and uses it in conjunction with a forward solution for the dipoles at each 3D brain voxel (of a specified size) to construct optimum spatial filters. The spatial filtering suppresses interference of unwanted signals from other locations.

For source estimation, the pre-ICA data were used. Data were band filtered (using the SAM default IIR filter) for each participant and condition and frequency band. Covariance matrices, and subsequently SAM weights, were computed for each 5 mm cubic voxel using the data from the two conditions participating in each signal change calculation, for each frequency-band-filtered time-series data. For each voxel, the data were multiplied by the weights, thus creating “virtual sensor” time-series, which were then transformed via FFT to the frequency domain, thus deriving power values. The next step involved calculating, for each frequency of each sensor of each participant, a power signal (SC) metric, for estimating activity differences between contrasted conditions. For normalization, SC was computed using a log ratio. More specifically, for sensor *S*, frequency *f*, and power values of conditions *A* and *B*, *SC*[*S*(*f*)] = *log*(*A/B*). Each participant's SC values for each of the comparisons were then collapsed across all sensors, and averaged across the frequency bands specified above in the sensor-level analysis.

To facilitate group analysis, head models were constructed by co-registering each participant's SAM volume to a previously obtained MRI scan (T1-weighted anatomical images acquired with high-resolution 1-mm slice thickness, obtained by Aviva Berkovich-Ohana, by means of a 3T Trio Magnetom Siemens scanner located at the Weizmann Institute of Science, Rehovot, Israel), based on the position of the fiduciary markers established during the digitization phase. Each participant's MRI and its co-registered SAM volume were then transposed into a common Talairach anatomical space (Talairach and Tournoux, 1988). Voxel-level group statistics, for each comparison and frequency band, were conducted using one-sample *t*-tests against the null hypothesis that the SC measures came from a continuous, normal distribution with a zero mean, and corrected for multiple comparisons based on a Monte Carlo simulation of random noise distribution (using AFNI's 3dClustSim module) (Forman et al., 1995).

## Results

### MEG sensor-level results

**Figure 3:**
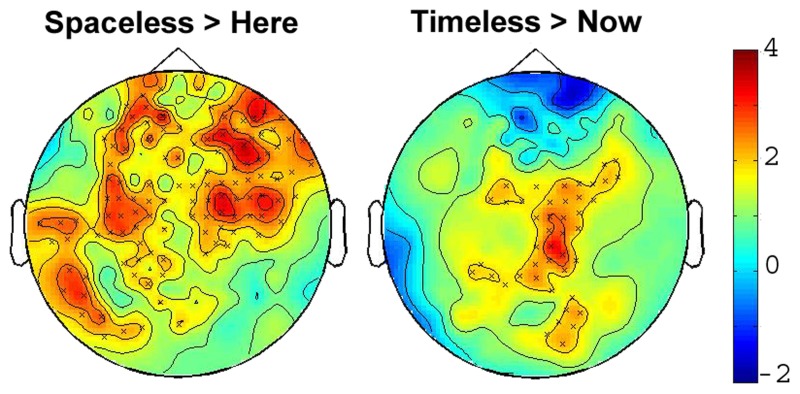
**2D scalp maps of theta power SC contrasts**. 2D topographic representations of significant sensor-level theta power (4–8 Hz) SC for the Timelessness vs. Now (**Right**), and Spacelessness vs. Here (**Left**). Crosses on the map represent significant clusters; color bar scale indicates *t*-values.

We first tested for significant differences in power between the “Here” and “Now” conditions, which were considered to be the baseline states for the other conditions. Importantly, there were no significant differences between them in any of the four frequency bands tested. Testing the “Spacelessness” vs. “Here” and the “Timelessness” vs. “Now” contrasts, we found clusters of significant differences only within the theta band (*p* = 0.014, and *p* = 0.049, respectively). Figure 3 provides 2D topographic representations of the sensor-level *t*-values for these two significant contrasts. As could be expected, the two contrasts evidence a different topography. The significant clusters for “Spacelessness” vs. “Here” occur predominantly over bilateral central-frontal electrodes, while “Timelessness” vs. “Now” shows central and right lateralized theta activity. However, there is an overlapping region (right central), which is further explored at the source level.

### MEG source localization estimates

To examine the neural activity underlying the conditions of “Timelessnes” and “Spacelessness,” “Timelessness” was compared to “Now,” and “Spacelessness” was compared to “Here.” Although the sensor-level analyses guided our source localization toward the theta frequency, we validated these results by searching for an overlap between the time and space comparisons over all frequency bands. Overlapping clusters were found only within the theta band, indicating increased activity. While not being the main goal of this study, we nevertheless report the “Timelessness” and “Spacelessness” activity patterns, before focusing on their overlapping activity.

**Figure 4:**
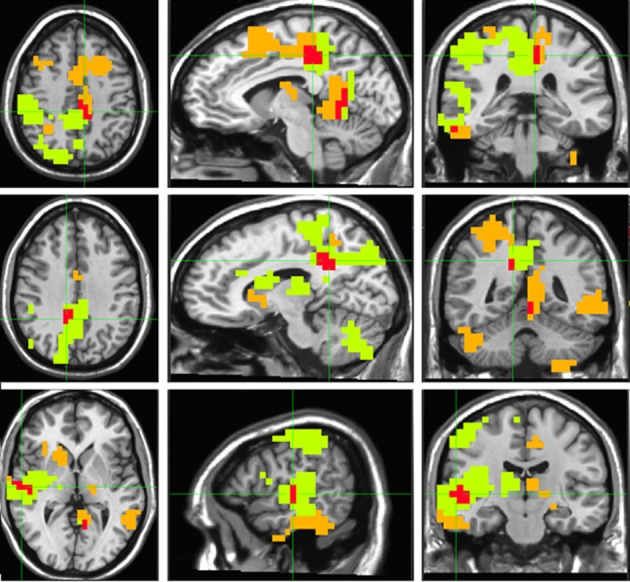
**Beamforming source estimates for the overlap between Timelessness vs. Now and Spacelessness vs. Here contrasts in the theta (4–8 Hz) frequency**. Axial, sagittal and coronal views (left to right) of group (*n* = 11) SAM pseudo-*F* source estimates overlayed on the Colin template. Note that in all images right and left sides are crossed. Green, orange, and red indicate Timelessness, Spacelessness and overlap between conditions, respectively. **Top**: left cingulate/precuneus and culmen (clusters 2 and 4, respectively, Table 3); **Center**: right cingulate/precuneus (cluster 1, Table 3); **Bottom**: right superior temporal gyrus (cluster 3, Table 3).

“Timelessness” vs. “Now” (Figure 4, Table 2) showed mostly right-lateralized (88%) theta activity spanning several regions, including right motor areas [postcentral gyrus and middle frontal gyrus (MFG)], parietal lobule, thalamus, basal ganglia, bilateral cerebellum, right temporal gyrus, right insula, right somatosensory and bilateral medial posterior cingulate cortices (PCC, including precuneus and cuneus). Spacelessness vs. Here showed theta activity which was bilaterally distributed (54% right hemisphere), over several regions (Figure 4, Table 2). It included bilateral PCC (with precuneus), bilateral cerebellum, bilateral parahippocampus, right basal ganglia, bilateral temporal gyrus, left thalamus, right postcentral gyrus, MFG right parietal lobule, and a small portion of the right insula. The “Timelessness” and “Spacelessness” conditions overlapped at the posterior part of the right superior temporal gyrus (STG), left cerebellum, and bilateral posterior cingulate cortex and adjacent precuneus (PCC/Prc) (Figure 4, Table 3).

**Table 2:**
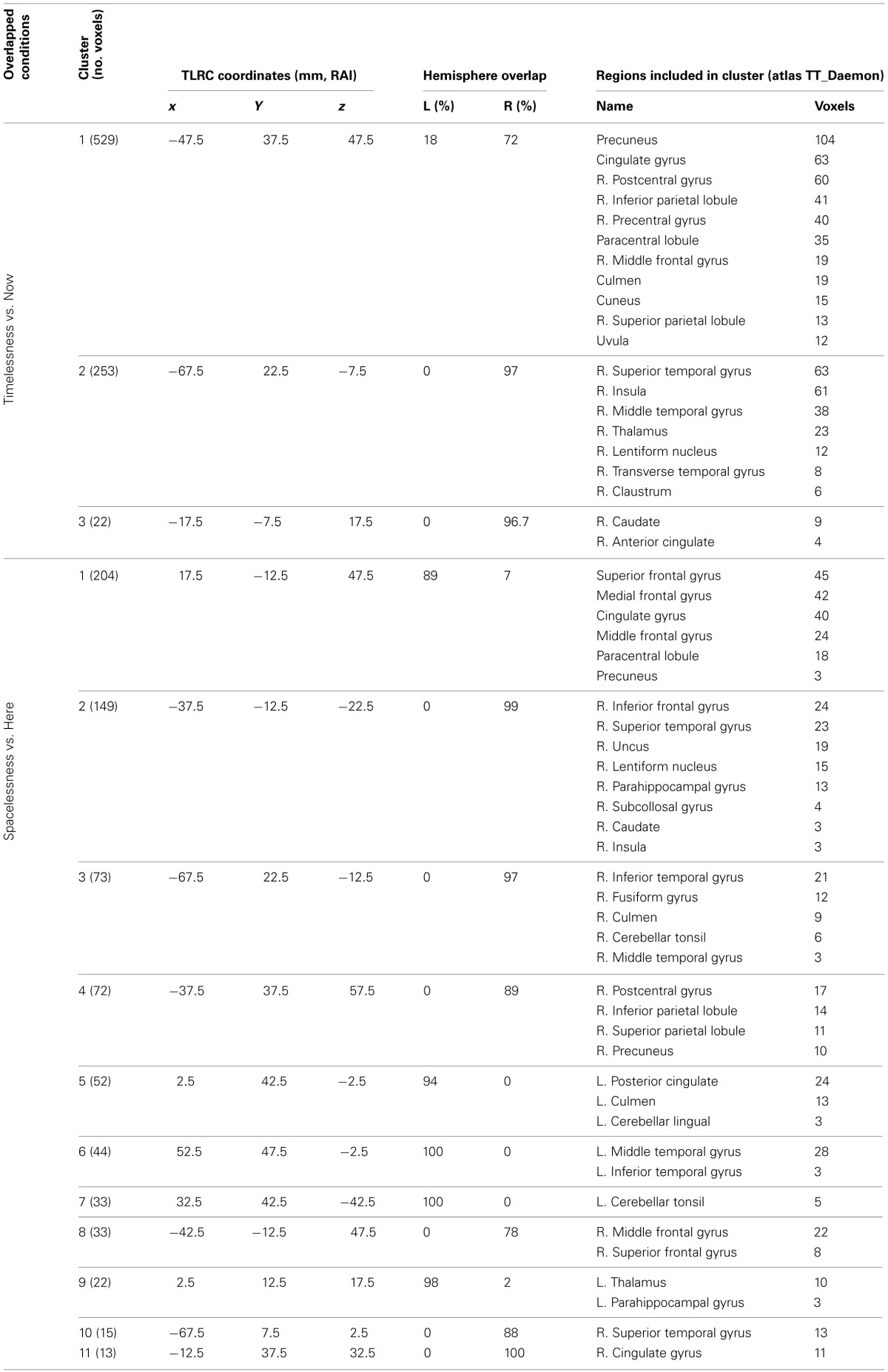
**Beamforming solutions for the contrasts Timelessness vs. Now and Spacelessness vs. Here in the theta (4–8 Hz) frequency band (*n* = 11)**.

Information supplied includes number of voxels in each cluster, center of cluster characteristics, hemispheric overlap, brain regions, and number of voxels in the region. Only clusters >2 voxels are presented. The Afni-supplied TT Daemon atlas was used. Due to poor resolution and signal leakage to non-brain regions, overlap percentages do not always add up to 100%.

**Table 3:**
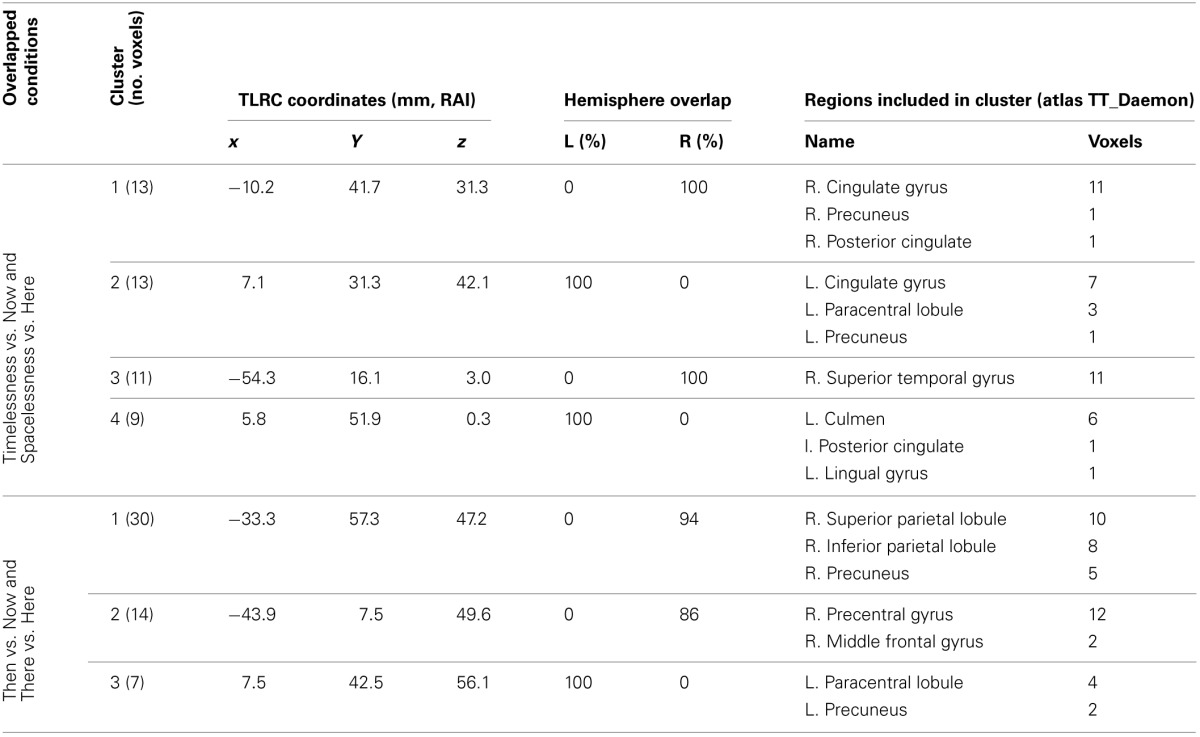
**Beamforming solutions for the overlapping contrasts between Timelessness/Spacelessness and Then/There in the theta (4–8 Hz) frequency band (*n* = 11)**.

Information supplied includes number of voxels in each overlapping cluster, center of cluster characteristics, hemispheric overlap, brain regions, and number of voxels in the region. Only clusters >1 voxels are presented. The Afni-supplied TT Daemon atlas was used. Due to poor resolution and signal leakage to non-brain regions, overlap percentages do not always add up to 100%.

**Figure 5:**
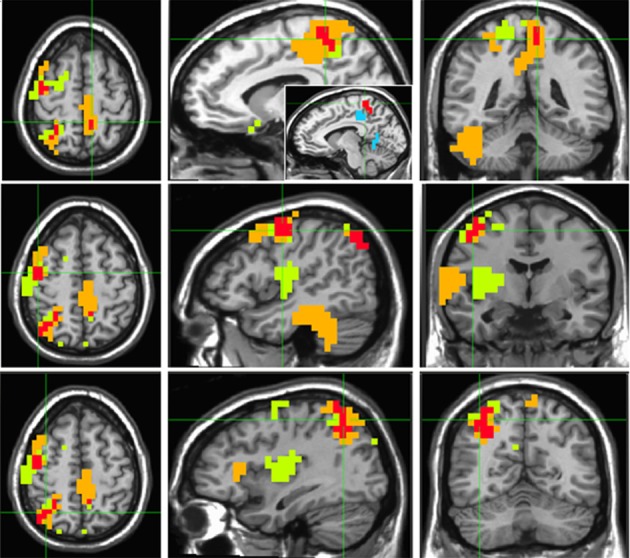
**Beamforming source estimates for the overlap between Then vs. Now and There vs. Here contrasts in the theta (4–8 Hz) frequency**. Axial, sagittal, and coronal views (left to right) of group (*n* = 11) SAM pseudo-*F* source estimates overlayed on the Colin template. Note that in all images right and left sides are crossed. Green, orange and red indicate Then, There and overlap between conditions, respectively. **Top**: left paracentral lobule and precuneus (cluster 3, Table 3). In the sagittal view, the same cluster (red) is compared with left cingulate/precuneus activity (cyan) for the Timelesness/Spacelessness overlap (cluster 2, Table 3). Note the clear separation between the superior cluster found in the Then/There overlap, and the inferior cluster found for the Timelesness/Spacelessness overlap; **Center**: right precentral and middle frontal gyrus (cluster 2, Table 3); **Bottom**: posterior parietal lobule (cluster 1, Table 3).

In order to control for attention, memory or imagination processes, we contrasted, in source space, the “Then” vs. “Now” and “There” vs. “Here' conditions, and then looked for an overlap between them. As before, while focusing on the theta rhythm, we checked for overlapping regions over all frequency bands. Overlapping clusters were found mostly within the theta band, with the only exception being one alpha band cluster localized exactly (same 10 voxels) over theta cluster no. 1, on the right superior parietal lobule (Figure 5, Table 3). The two contrasts overlapped in three clusters, which included four main regions: Right posterior parietal lobule (PPL), right precentral and MFG, and bilateral precuneus (Figure 5, Table 3). Yet, there were regions which did not overlap between the two contrasts (Table 4). These included the right superior temporal gyrus (STG), which although activated in both contrasts, did not overlap. Additionally, only “Then” vs. “Now” activated the right insula, and only “There” vs. “Here” activated the right anterior cingulate and right lateral cerebellum.

**Table 4:**
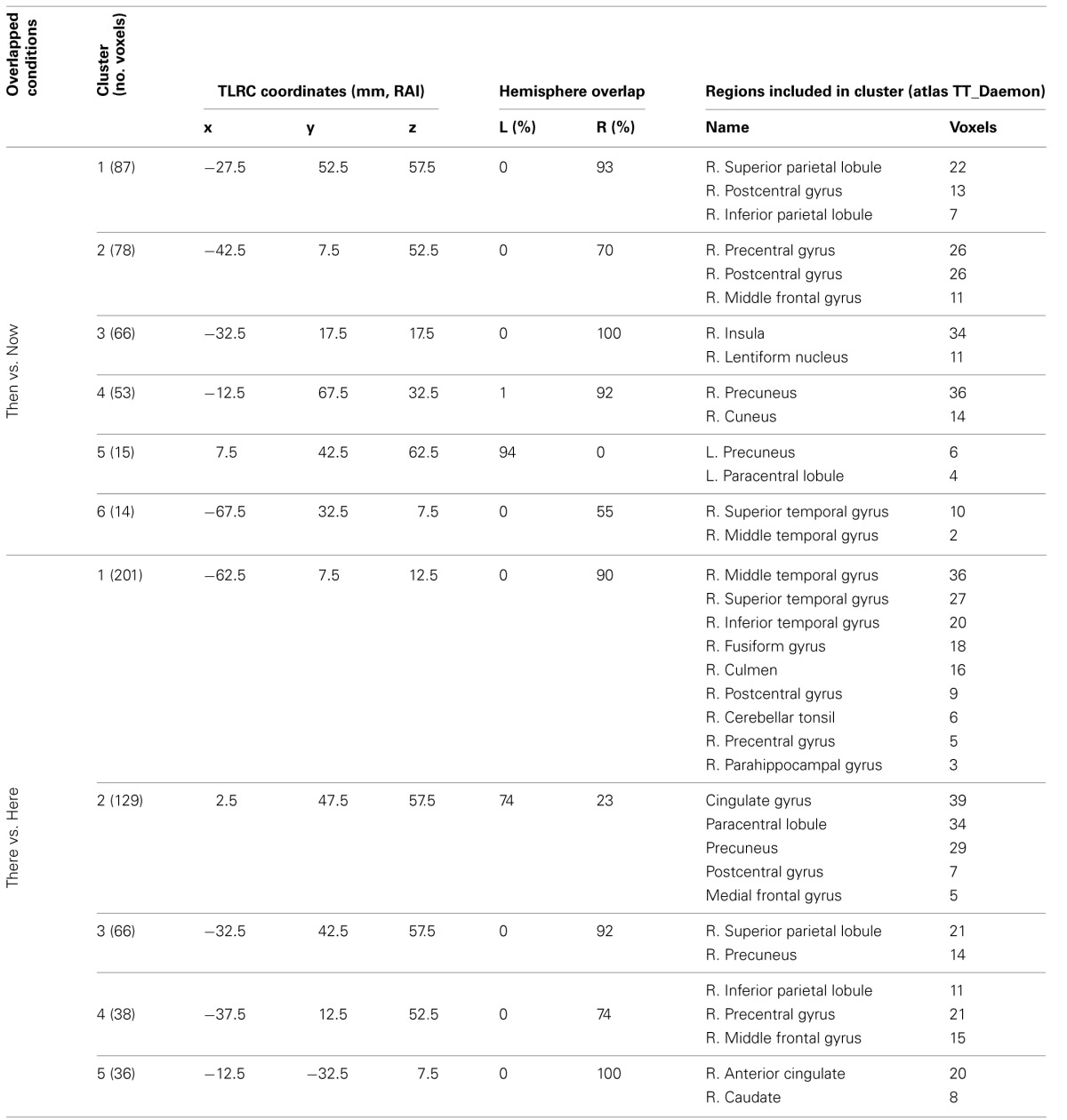
**Beamforming solutions for contrasts Then vs. Now and There vs. Here in the theta (4–8 Hz) frequency band**.

Information supplied includes number of voxels in each cluster, center of cluster characteristics, hemispheric overlap, brain regions, and number of voxels in the region. Only clusters >2 voxels are presented. The Afni-supplied TT Daemon atlas was used. Due to poor resolution and signal leakage to non-brain regions, overlap percentages do not always add up to 100%.

### Neurophenomenologically-guided MEG analysis

**Figure 6:**
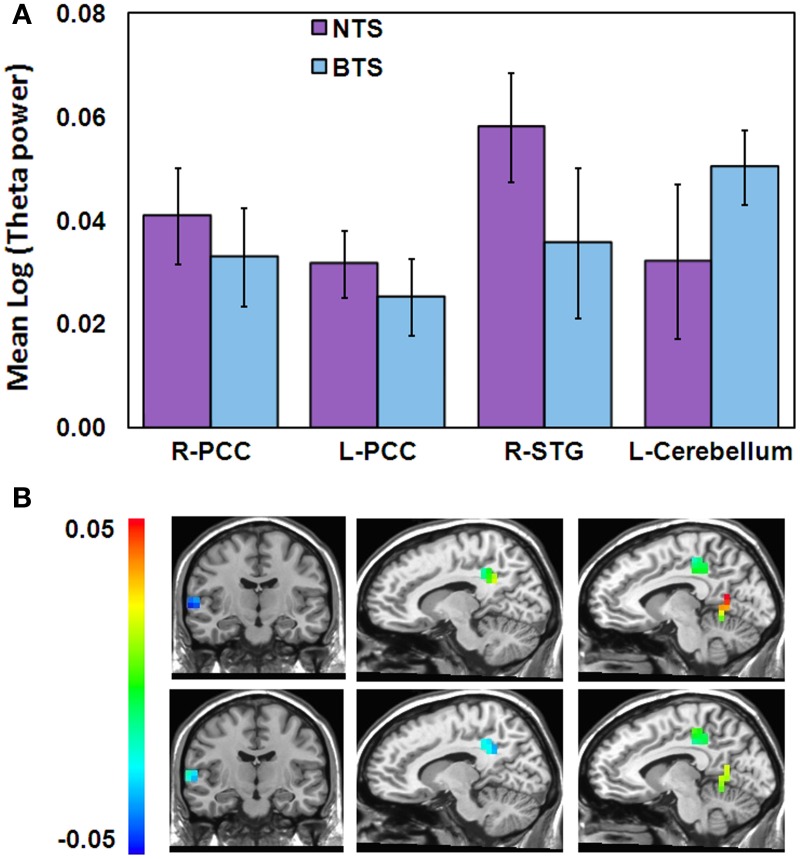
**Differences in theta activity between the BTS (*n* = 5) and NTS (*n* = 6) groups over 4 overlapping ROIs. (A)** Group × Cluster interaction. BTS, both time and space; NTS, Not both Time and Space; **(B)** Spacelessness (top) and Timelessness (bottom) group theta differences within the 4 clusters, calculated as BTS minus NTS. Color scale denotes log(theta power) values. Left column—right superior temporal gyrus (STG); Middle column—right posterior cingulated cortex (PCC); Right column—left PCC and cerebellum clusters.

To study possible differences between the BTS and NTS groups, we derived for each participant the mean theta activity value within each of the four clusters of overlap between the “Timelessness” and “Spacelessness” conditions. On these values, we ran a Three-Way ANOVA with one grouping factor (BTS, NTS) and repeated measures on Condition (Timelessness, Spacelessness) × Cluster (R-PCC, L-PCC, R-STG, cerebellum). There was no main effect for Condition, Cluster or Group. However, we found a significant Group × Cluster interaction [*F*_(3, 27)_ = 2.85, *MSE* = 0.001, *p* < 0.05]. As can be seen in Figure 6, the BTS group exhibited lower R-STG and higher L-cerebellum theta activity compared to the NTS group.

**Figure 7:**
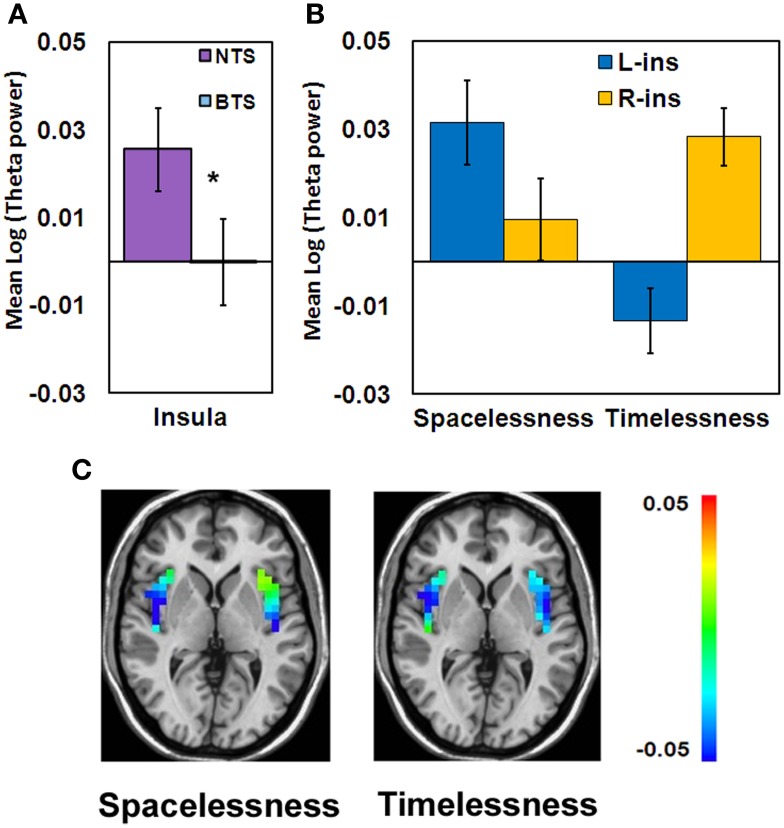
**Theta activity differences between the BTS (*n* = 4) and NTS (*n* = 5) groups over insula. (A)** Group main effect. ^*^p < 0.05. BTS, both time and space; NTS, Not both Time and Space. **(B)** Condition × Hemisphere interaction. R, right; L, left. **(C)** Group differences in theta activity, calculated as BTS minus NTS. Color scale denotes log(theta power) values.

While the insula showed theta activity during both “Timelessness” and “Spacelessness,” the activity pattern was not overlapping. As a result, this region does not appear as an overlapping cluster. However, in order to test the hypothesis that group differences in bodily boundaries are related to insula activity, as the insula is a major interoceptive regions (Craig, 2002), we defined the insula anatomically as an ROI, and then calculated the theta activity value within the bilateral insula for the overlap between the “Timelessness” and “Spacelessness” conditions. On these values, we ran a Three-Way ANOVA on Group (BTS, NTS) × Condition (Timelessness, Spacelessness) × Hemisphere (L, R). Two outliers with very high values (above two standard deviations from the group mean) for the “Spacelessness” condition, one from each group, were excluded. We found a Condition × Hemisphere interaction [*F*_(1, 7)_ = 5.52, MSE = 0.002, *p* < 0.05], with “Spacelessness” being left-lateralized, and “Timelessness” being right-lateralized (the latter being significant [p < 0.05, Bonferroni-corrected, *post-hoc t*-test]) (Figure 7A). Importantly, we found a main effect for Group [*F*_(1, 7)_ = 6.12, MSE = 0.006, *p* < 0.05], with the BTS group showing lower overall insula theta activity compared to NTS (Figures 7B,C).

**Figure 8:**
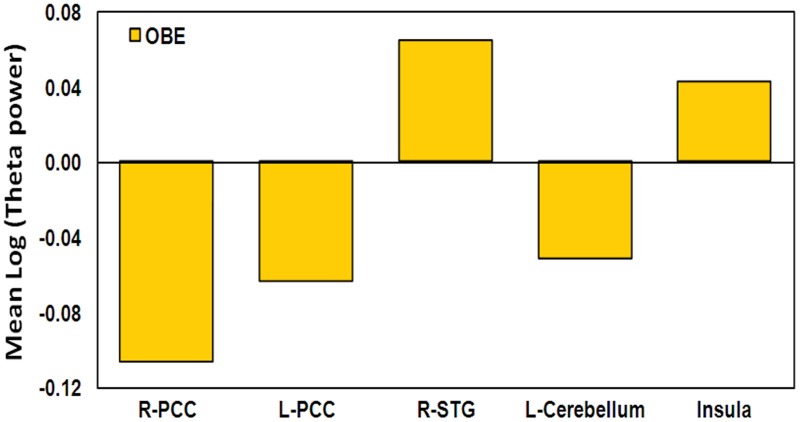
**OBE participant activity in the ROIs**. Mean log theta power during the Timelessness and Spacelessness conditions, in the selected ROIs.

For comparison purposes, we show the mean theta activity value within each of the four clusters of overlap between the “Timelessness” and “Spacelessness” conditions for the one participant who reported an OBE (Figure 8). In comparison with the BTS and NTS groups, the OBE participant exhibited much lower bilateral PCC and left cerebellar values. Right MTG and total insula values were slightly higher than the NTS group.

## Discussion

### MEG sensor-level results

Significant differences in power between the “Spacelessness” vs. “Here” as well as the “Timelessness” vs. “Now” contrasts were found only within the theta frequency. Both contrasts showed maximal theta power over the right hemisphere. This result is in line with accumulating evidence from animal and human studies, showing that theta activity is tightly related to space and time processing, i.e., encoding and retrieval (recently reviewed by Hasselmo and Stern, 2013). This is also in agreement with the notion that there is a right-hemisphere specialization for space and time processing (Rao et al., 2001; Ellison et al., 2004; Oliveri et al., 2009a,b; reviewed by Walsh, 2003).

The production of theta with eyes closed is a well-known accompaniment of states of deep relaxation such as stage 1 sleep, meditation and hypnosis (Vaitl et al., 2005). Gruzelier (2009) reviews EEG-neurofeedback (NF) training studies for increasing theta activity, showing wide behavioral effects, including increased creativity, heightening psychological integration, relief from anxiety and depression and resolved post traumatic stress syndrome. Phenomenologically, participants in this EEG-NF protocol reported increased theta “to be associated with a deeply internalized state and with a quieting of the body, emotions, and thought” (Gruzelier, 2009, p. 102). Based on the findings that theta oscillations play a critical role in the coupling and integration of widely distributed neural circuits (Von Stein and Sarnthein, 2000), as well as the EEG-NF results, Gruzelier (2009) proposed that the wide ranging behavioral correlates of theta result from theta's role in mediating distributed circuitry in the brain, relating the concepts of psychological integration (integrative experiences leading to feelings of psychological well-being) and neural integration.

The “Timelessness” and “Spacelessness” conditions represent alterations in the sense of time and space, akin to those reported in various meditative practices, and can be compared to previously reported theta topography in studies of meditative states. The increased frontal-central theta power (Figure 3) is in accord with ample meditation studies, reporting increased theta activity, mostly over frontal-central sites (Hebert and Lehmann, 1977; Aftanas and Golocheikine, 2001; Kubota et al., 2001; Faber et al., 2004; Cahn and Polich, 2006; Slagter et al., 2009; Baijal and Srinivasan, 2010). Increased theta in meditation studies has been interpreted as reflecting internalized attention (Cahn and Polich, 2006), space and time processing (Baijal and Srinivasan, 2010), as well as being related, especially when manifesting as theta bursts, to deep meditative states, feelings of blissfulness and low thought content, and a sense of integration (Kasamatsu and Hirai, 1966; Hebert and Lehmann, 1977; Aftanas and Golocheikine, 2001).

The results are also in agreement with the junction-point-model hypothesis proposed by Travis (1994). Briefly, Travis proposes that bursts in the 7–9 Hz band underlie the state of transcendental consciousness (Travis, 1994), which is “the least excited state of mental activity,” unbounded by a sense of time and space (Alexander et al., 1987). Travis suggests that transcendental consciousness underlies other forms of consciousness, and can be seen especially during transitions between different states of consciousness. He then provides support for his hypothesis by showing increased 7–9 Hz activity during the transition between waking, NREM-sleep, and REM-dreaming. Similar theta activity has also been reported during Transcendental Meditation (TM); and in TM practitioners, it also unusually accompanies sleep, a state called witnessing sleep (Travis, 1994). This state differs from lucid dreaming by increased “separateness” and reduced dream control (Alexander, 1988). The junction-point model ties all these states as windows to an underlying field of transcendental consciousness, related to alterations in the sense of time and space, and a sense of boundlessness.

Taken together, the literature on theta in neurofeedback and meditation suggests that this is the optimal bandwidth for integration and synthesis across neural regions. As Hunt (2007, p. 226) argues, “widespread EEG theta would appear to be the level of activation which affords a maximized coherence across the widest possible neural areas….” This interpretation aptly fits the phenomenology of the “Timelessness” and “Spacelessness” states, which include many descriptions of integration (Table 1): “The purest form of the sensation was there, but un-restricted. There was relaxation and widening” and “There was a sense of open space without the bodily dimension” (participant no. 11); “It was a sense of spaciousness, boundlessness… there was no clarity where the center is and where is the periphery. There was no quality of border” (participant no. 12); “There was relaxation and letting-go, emptiness, experience of bliss, quiet, wide” and “The metaphor is an amoeba, everything spreads. A sense of nothingness, emptiness. A sense of expansion” (participant no. 5); “I lost discrimination between different body parts. It felt pleasant, like a huge hammock. A sense of expansion” (participant no. 14); “The center of space became endless, without a reference point in the middle. There was a sense of floating in a sea of being… Everything was a part of one conscious experience. The dichotomy between subject and object dissolved… Everything was a part of a singular event. The experience of the body faded” (participant no. 4). Indeed, Faber et al. (2012) discuss the question, “Why are there so few systematic reports on subjective experience during meditation,” and emphasize that “it could be very useful in sorting out brain states of different cogitations” (p. 262). In this study, we have shown how such reports can be utilized in a productive way, both to aid in interpreting brain activity during these unique states, but also to learn more about these states, from the systematic self observations of our meditators. One does not have to rely on the ingestion of a hallucinogen such as *ayahuasca*, to uncover what Bresnick and Levin (2006) term “profound alterations of temporal-spatial experiences including expansive space and slowed time.” These experiences can be found in long-term meditators. Whether the meditators in this study are more aware of what Travis (1994) terms “an underlying, undifferentiated field,” wherein presumably there is an alteration in the experience of both space and time, is a metaphysical issue—and not one that we can resolve using the present experimental protocol. Nevertheless, as Hunt (2007, p. 226) has suggested, “advanced meditation involves an attunement to a background field of consciousness, whose increased meditative access seems to be correlated with an unusually coherent EEG in the theta bandwidth.” What might be considered to be the *psychedelic* effects invoked by systematically observing (or, becoming sensitized to) one's experience of both space and time—sometimes resulting in spacelessness and/or timelessness—might, in turn, be actually the externalization of this background field of consciousness (Hunt and Chefurka, 1976). We cannot make a conclusive case here for this; we can, however, provide a portal for future research, building on the protocol explored here.

### MEG source localization estimates

#### “Then” and “there” (control) conditions, and their overlap

The “Then” vs. “Now” and “There” vs. “Here” contrasts served as control conditions for ruling out autobiographic memory and imagination processes, respectively, as well as attentional processes, which might have taken place during the “Timelessness” and “Spacelessness” target conditions. In both contrasts, only increased theta activity was detected, mostly right-lateralized (Tables 3, 4). Within the context of the hypothesis that time can be represented along a left-to-right oriented mental time-line, and based on psychophysical and neuroimaging studies, it has been suggested that the right hemisphere entails the representation of the past and the left hemisphere the representation of the future (Szpunar et al., 2007; Oliveri et al., 2009a). The right hemisphere dominance in the “Then” vs. “Now” contrast is in line with this, as participants were recalling past events.

The two contrasts overlapped in three clusters, which included four main regions: Right PPL, right precentral and MFG, and bilateral precuneus (Figure 5, Table 3). These results are in line with fMRI studies of mental traveling to the past, reporting bilateral PPL activation (Arzy et al., 2009) and left precuneus and MFG activation (Szpunar et al., 2007). Moreover, the results support our hypothesis of episodic memory network activation in the control conditions, as the right PPL and MPG were shown to be involved in episodic memory performance (Rajah et al., 2011). Similarly, the precuneus is a region involved with autobiographic memory (Cabeza and Nyberg, 2000; Rugg et al., 2003), as well as mental imagery (Lundstrom et al., 2003). Altogether, the overlapping pattern of the “Then” vs. “Now” and “There” vs. “Here” conditions reveals a largely right-lateralized network specialized in episodic memory, as well as mental imagery of one's body.

#### Alteration in the experience of time

The “Timelessness” vs. “Now” (Figure 4, Table 2) contrast revealed theta activity in a right-lateralized network of parietal, temporal and insular cortical regions, as well as the basal ganglia, thalamus and cerebellum. Specifically, these regions included the motor areas (postcentral gyrus) spreading into supplementary motor area—SMA (MFG), parietal lobule, thalamus, and basal ganglia. This is consistent with previous prospective timing studies suggesting that fronto-striatal circuits consisting of recurrent loops between SMA, basal ganglia and thalamus are critical for the processing of duration (Coull and Nobre, 1998; Ferrandez et al., 2003; Coull, 2004; Wittmann, 2009), as well as with neuroimaging of temporal task performance documenting recruitment of the right posterior parietal cortex (Coull and Nobre, 1998; Walsh, 2003; Oliveri et al., 2009a). Theta activity was also found in the bilateral cerebellum, in accord with TMS (Tomlinson et al., 2013) and PET (Coull and Nobre, 1998) studies showing a cerebellar role in time perception and representation (Salman, 2002), as well as over the right temporal gyrus, a region shown previously to be involved in time production in lesion (Noulhiane et al., 2007) and TMS (Bueti et al., 2008) studies. Finally, theta activity was seen in the right insula, right somatosensory and bilateral medial posterior cingulate cortices, a network involved in somatic information processing. The interoceptive insula has been previously suggested to be responsible for the perception of duration (Craig, 2009a,b; Wittmann, 2009; Wittmann et al., 2010). Indeed, an fMRI study of MM practitioners showed that attending to the present moment was accompanied by activation in the right insula as well as the somatosensory cortex (Farb et al., 2007).

Altogether, the regions recruited during the “Timelessness” condition were previously related to either momentary processing of time, or interoception. This is in accord with our hypothesis that alteration in the expereince of time is related to an altered sense of body.

#### Alteration in the experience of space

“Spacelessness” vs. “Here” showed theta activity which was largely bilaterally distributed, over several regions (Figure 4, Table 2). Activated regions included bilateral cerebellum—known to regulate balance via processing of vestibular information (Timmann et al., 2010); bilateral parahippocampus, known to subserve spatial computation and learning (Aguirre et al., 1996); and right basal ganglia, sensitive to spatially-related behavioral conditions (Lavoie and Mizumori, 1994). Additionally, bilateral temporal gyrus, left thalamus, right postcentral gyrus, MFG, bilateral frontal cortices and right parietal lobule (IPL) were activated, all related to space processing (Halligan et al., 2003; Hagler and Sereno, 2006; Silver and Kastner, 2009). Additionally, the bilateral PCC and a small portion of the right insula, both related to interoception (Damasio and Meyer, 2009), were activated. Moreover, the PCC is involved in processing vestibular information (Wiest et al., 2004) supporting its major role in navigation of the body in space (Vogeley et al., 2004; Vogt and Laureys, 2005).

Altogether, the regions showing theta activity during the “Spacelessness” condition were previously related with some form of spatial processing (summarized by Iacoboni et al., 1997), or with interoceptive processing. This, again, is in line with our hypothesis that the alteration in the experience of space is related to an altered sense of body.

#### Shared alterations in the experience of time, space, and body

The “Timelessness” and “Spacelessness” conditions overlapped in four clusters (Figure 4, Table 3): bilateral cingulate cortex/precuneus (PCC/Prc), right temporoparietal junction (TPJ) and left cerebellum. In line with our hypothesis, this pattern of theta activity was different from the overlap pattern between the control conditions of “There” and “Then,” which showed activity in regions related to mental imagery and episodic memory. We argue that this overlap pattern is predominantly related to alterations in the experience of the body. Subsequently, we provide support for this argument, relating the overlapping regions with bodily processing.

The PCC/Prc plays a central role in consciousness (Cavanna and Trimble, 2006) as these regions differentiate patients in minimally conscious states from those in vegetative states (Laureys, 2005; Vogt and Laureys, 2005; Vanhaudenhuyse et al., 2010), and are deactivated during REM sleep, when participants experience vivid dreams (Alkire et al., 2008). In addition, these are key regions for bodily representation (Damasio, 1998, 1999; Damasio and Meyer, 2009) and vestibular processing (Wiest et al., 2004). Importantly, the PCC/Prc overlap for the “Timelessness” and “Spacelessness” conditions clearly differs from the left precuneus cluster found in the “There” and “Then” overlap (see relative activity in Figure 5), by being relatively inferior, anatomically. A recent fMRI study of emotional processing (Immordino-Yang et al., 2009) proposes a different role for inferior and superior parietal medial cortex, suggesting the first to be related to interoceptive processing, and the second to musculoskeletal processing. Consistent with that emotional processing study, our data suggest a functional subdivision in the parietal medial cortex regarding spatio-temporal processing. While the “There” and “Then” conditions overlapped in a more superior position, strongly related anatomically with the lateral parietal cortex (Parvizi et al., 2006), which is activated in episodic memory and imagination, the “Timelessness” and “Spacelessness” conditions overlapped at an inferior position, strongly related with interoception (Damasio and Meyer, 2009).

The caudate part of the STG is encompassed within the temporo-parietal junction (TPJ). The TPJ is a multimodal association cortex, integrating thalamic, visual, auditory, somatic, and limbic areas (Decety and Lamm, 2007). It is also a key region for multisensory body-related self-processing, related to a first-person perspective; damage to this area can produce a variety of disorders associated with bodily awareness (Blanke and Arzy, 2005). Recent findings emphasize the role of the right TPJ in abnormal vestibular processing, as during OBEs (Blanke et al., 2004), and Near-Death Experience (NDE) characterized by an altered sense of time, and sensations of lightness and void (Blanke and Dieguez, 2009).

The observed cerebellar activity is within the midline culmen. This area is the vestibulo-cerebellum (Barmack, 2003). Several studies point to the role of the cerebellum in altered sense of body. In a PET study where OBEs were repeatedly elicited during stimulation of the right TPJ in a patient in whom electrodes had been implanted to suppress tinnitus (De Ridder et al., 2007), a strikingly similar pattern of activity to our overlapping pattern was found. Activity during OBE was seen at the right TPJ, right precuneus and superior vermis of the cerebellum. In another single-subject study (Schutter et al., 2006), the participant reported that after cerebellum rTMS, but not after sham and occipital stimulations, she experienced her body falling/drifting sidewards and even out of the chair.

To summarize, we show that an altered experience of time and space involves theta activity in regions related to a sense of the body. These findings support the phenomenologically reported spaciousness, a sense of alteration in the regular bodily boundaries. The type of altered sense of body found in this study include states where the sense of self becomes diffused and “spills out” of the body boundaries (participants no. 1, 2, 4, and 5), or simply disappears (participants no. 12, 13, and 14), and not just cases where the self simply changes its location, as in an OBE (participant no. 8).

#### Neurophenomenologically-guided analyses

The participants were split into two groups based on the phenomenology of their reported experience of time and space. Interestingly, the both-time-and-space (BTS) group showed lower right TPJ and insula theta activity, accompanied by higher cerebellar theta activity, compared to the not-both-time-and-space (NTS) group. As all these regions are related to bodily processing and body schema, this shows that the BTS group activates regions involved in bodily processing differently than the NTS group. This might lead to their heightened alteration in sense of bodily boundaries, which is in turn related to their heightened alterations in the experience of time and space.

In comparison, the OBE participant manifested a markedly different pattern of theta activity compared to both the NTS and BTS groups (Figure 8). This might explain the preserved sense of body (though perceived as floating in space), “I was with my body, just floating in outer space. The body was identical, I wasn't floating as consciousness, but as a body” (Table 1). Here we see a different form of an altered sense of body—without loss of boundaries (not along the body—boundary continuum). This was expressed in this participant's very different bilateral PCC theta power values, which evidenced a reduction in theta activation, rather than an increase (as with the other participants). These distinctions highlight the utility of phenomenology in guiding neuroscientific analyses.

#### Summary of the MEG source localization estimates

The results obtained from the MEG source localization estimates provide evidence that alterations in the sense of time and space: (1) do not rely on memory and mental imagery; and (2) are related to an altered sense of body.

In relation to the sensor level results (section MEG Sensor-Level Results) pointing to the critical role of the theta band in such experiences, we suggest that theta activity in cortical regions related to bodily processing results in experiences of an altered sense of the body, of time and of space, where the different sensory modalities integrate, and sensorial boundaries decrease. The neurophenomenologically-guided analyses (section Neurophenomenologically-Guided Analyses) provide further evidence that different phenomenological experiences result in significantly different levels of theta activity within these cortical regions, which are related to bodily processing.

### Limitations of the study

This study has a number of limitations that should be taken into consideration. First, sample size was rather small after exclusion of five of the participants. The 11 remaining participants were further divided into two sub-groups, resulting in very small group sizes. Second, the study design was fixed, as opposed to counterbalanced, between the Time and Space sessions. The verbal ratings of success of the participants were lower for “Timelessness” compared to “Spacelessness.” This might be due to the study design, as the two conditions were studied in that order, which makes it impossible to dissociate perceived task difficulty from increasing depth of state due to the previous session, or due to increasing acquaintance with the experimental setup. Thus, all results reported here should be considered preliminary, and warrant replication.

## Conclusions

This study illustrates the utility of employing experienced contemplative practitioners within a neurophenomenological setup for scientifically characterizing self-induced altered spatio-temporal experiences. The results reported here support our hypotheses that an altered experience of time and space is related to an altered sense of body, by showing that: (1) “Timelessness” and “Spacelessness” conditions overlap in a neural network related to alterations in bodily processing. This was shown to be distinct from mere memory and imagination processes; and (2) phenomenologically-guided neural analyses yielded further insight, by enabling us to dissociate different levels of an altered sense of the body. Additionally, our results underscored the specificity of theta activity, and emphasize theta's unique role in altered experience of time, space and the body. Taken together, the results reported here support previous suggestions of the psychological integrative role of the theta band (Hunt, 2007; Gruzelier, 2009), and provide further understanding of deep meditative states, reported frequently to invoke enhanced theta activity.

## Author contribution

Joseph Glicksohn, Aviva Berkovich-Ohana, and Abraham Goldstein sponsored the study; Abraham Goldstein provided research facilities; Aviva Berkovich-Ohana and Joseph Glicksohn designed, while Yair Dor-Ziderman and Aviva Berkovich-Ohana ran the experiment; Yair Dor-Ziderman analyzed MEG data; Aviva Berkovich-Ohana analyzed phenomenological data and Aviva Berkovich-Ohana, Joseph Glicksohn, and Yair Dor-Ziderman wrote the paper.

### Conflict of interest statement

The authors declare that the research was conducted in the absence of any commercial or financial relationships that could be construed as a potential conflict of interest.
